# Distributed Denial of Service Attack Detection in Network Traffic Using Deep Learning Algorithm

**DOI:** 10.3390/s23208642

**Published:** 2023-10-23

**Authors:** Mahrukh Ramzan, Muhammad Shoaib, Ayesha Altaf, Shazia Arshad, Faiza Iqbal, Ángel Kuc Castilla, Imran Ashraf

**Affiliations:** 1 Department of Computer Science, University of Engineering & Technology (UET), Lahore 54890, Pakistan; mahrukh312@gmail.com (M.R.); shoaib@uet.edu.pk (M.S.); shazia.shoaib@uet.edu.pk (S.A.); faiza.iqbal@uet.edu.pk (F.I.); 2 Universidad Europea del Atlántico, Isabel Torres 21, 39011 Santander, Spain; angel.kuc@unini.edu.mx; 3 Universidad Internacional Iberoamericana, Campeche 24560, Mexico; 4 Universidad Internacional Iberoamericana, Arecibo, PR 00613, USA; 5 Department of Information and Communication Engineering, Yeungnam University, Gyeongsan 38541, Republic of Korea

**Keywords:** distributed denial of service attacks, denial of service attack detection, deep learning, network security

## Abstract

Internet security is a major concern these days due to the increasing demand for information technology (IT)-based platforms and cloud computing. With its expansion, the Internet has been facing various types of attacks. Viruses, denial of service (DoS) attacks, distributed DoS (DDoS) attacks, code injection attacks, and spoofing are the most common types of attacks in the modern era. Due to the expansion of IT, the volume and severity of network attacks have been increasing lately. DoS and DDoS are the most frequently reported network traffic attacks. Traditional solutions such as intrusion detection systems and firewalls cannot detect complex DDoS and DoS attacks. With the integration of artificial intelligence-based machine learning and deep learning methods, several novel approaches have been presented for DoS and DDoS detection. In particular, deep learning models have played a crucial role in detecting DDoS attacks due to their exceptional performance. This study adopts deep learning models including recurrent neural network (RNN), long short-term memory (LSTM), and gradient recurrent unit (GRU) to detect DDoS attacks on the most recent dataset, CICDDoS2019, and a comparative analysis is conducted with the CICIDS2017 dataset. The comparative analysis contributes to the development of a competent and accurate method for detecting DDoS attacks with reduced execution time and complexity. The experimental results demonstrate that models perform equally well on the CICDDoS2019 dataset with an accuracy score of 0.99, but there is a difference in execution time, with GRU showing less execution time than those of RNN and LSTM.

## 1. Introduction

The use of Internet technology is expanding rapidly, enabling hundreds of thousands of devices to perform online operations. The Internet is being widely embraced in different domains; it has expanded and is vulnerable to several attacks. Among such attacks, denial of service (DoS) and distributed DoS (DDoS) are the most frequently occurring attacks. There are many methods to launch DoS attacks. The primary goal of DoS and DDoS is to stop the services provided by applications to users by exhausting the network resources. DDoS attacks occur when the hosted server is targeted with a large number of irrelevant traffic by zombie devices [1].

**Figure 1:**
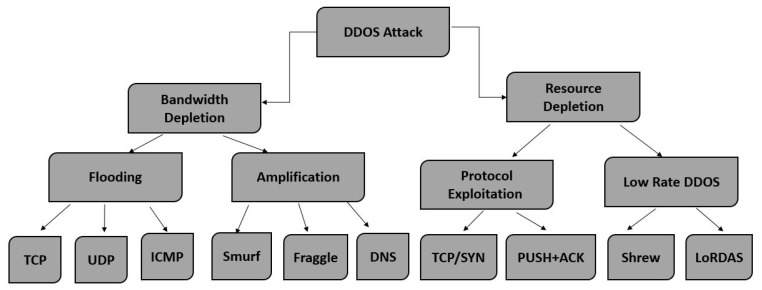
Categorization of DDoS attacks.

DoS and DDoS attacks are growing in strength and frequency. An average of 28.7 k attacks are launched every day. As per Neustar’s Cyber Threats and Trends Report, the frequency of DDoS attacks increased by 200% in the first six months of 2019, while the volume increased by 73% in 2018. It is predicted that by the end of 2023, the total number of DDoS attacks will be doubled compared to 2018, reaching up to 15.4 million. The Neustar’s Cyber Threats and Trends Report 2020 indicates that a 151% increase in the number of attacks was observed in June 2020, compared to 2019 [2]. In addition, there is a 192% increase in the largest attack size and an 81% increase in the maximum attack intensity. The attack volume has also increased to 12Gbps in June 2020, compared to 11Gbps in 2019 for the same period. Therefore, there is an increased need to develop a solution to detect DDoS attacks effectively and successfully [3,4]. Very well-known DDoS attacks are SYN, TCP, ICMP, UDP, HTTP, and DNS flood [5]. DDoS attack types and their sub-types are shown in Figure 1.

Several machine learning (ML) and deep learning (DL) models have been utilized for network attack detection. For example, decision tree (DT), logistic regression (LoR), linear regression (LR), Naive Bayes (NB), support vector machine (SVM), K nearest neighbor (KNN), random forest (RF), XGBoost, AdaBoosting, ResNet, artificial neural networks (ANNs), and convolutional neural networks (CNNs) are implemented using the CICDDoS2019 dataset to detect the DDoS attacks [6]. In addition, the CICIDS2017 dataset, KDD datasets, CAIDA 2007 dataset, IoT NI, BoT IoT, MQTT, MQTTset, IoT-23, IoT-DS2, and UNSWNB15 datasets are utilized for DDoS attack detection.

The CICDDoS2019 [6] dataset is a well-known dataset for analyzing the performance of ML and DL models for DDoS attacks. It contains real-time DDoS attacks from network traffic. The dataset contains a vast variety of DDoS attacks. There are 12 types of attacks available in the dataset, including ’DNS’, ’SNMP’, ’NTP’, ’WebDDoS’, ’MSSQL’, ’UDP’, ’LDAP’, ’NetBIOS’, ’SSDP’, ’PortScan’, ’UDP-Lag’, and ’SYN’. Many researchers used this dataset in their research to find the best features and the best model to detect DDoS attacks with minimum execution time and cost.

DL techniques are much better than ML techniques in terms of precision and accuracy, and can process huge amounts of data [7]. Recurrent neural networks (RNNs) are useful for large amounts of data and they use previous computation and current input for evaluation. RNN is useful when information is preserved with minimal loss. Long short-term memory (LSTM) and gated recurrent units (GRU) are a special form of RNN. The primary motivation for using LSTM and GRU is the retention of prominent information for later use in the system, which could work effectively in detecting both known and unknown attacks [5,8].

This study adopts the DL models for detecting DDoS attacks using the CICDDoS2019 dataset. DL models RNN, LSTM, and GRU are utilized for experiments. The dataset is preprocessed, involving several steps like data normalization, dealing with missing values and null values, transforming categorical values, label encoding, and feature selection. Feature selection is performed to select the top 20 features for obtaining better performance. Experimental results are presented concerning training and validation graphs, as well as accuracy, recall, precision, accuracy, F1 score, and execution time for binary and multi-classification [9].

Section 2 describes the related work for this study. Section 3 presents the proposed methodology, including the implemented models, selection of models, and parameter optimization. Results and discussion are given in Section 4. Finally, Section 5 concludes this study.

## 2. Related Work

This section presents previous work in the form of a comprehensive literature review. IT has gained significant popularity in the modern world. DoS and DDoS are the most prevalent attacks that compromise IT security. The primary objective of an attack is to disable victims’ devices and make them inaccessible to legitimate users. A large body of work can be found on network attack detection. For example, the research in [1] discussed the problems associated with DDoS attacks in the Internet of Things (IoT) devices. The perception layer, also known as the sensing layer, uses radio frequency identification (RFID) tags, global positioning system (GPS), wireless sensor network (WSN), Bluetooth, and cameras for attack detection. There have been eavesdropping and radio frequency (RF) jamming attacks at the perception layer. Flooding and reflection attacks are well-known network layer attacks. Signature wrapping attacks and flooding attacks are well-known types of middleware layer attacks. Reprogramming attacks and path-based DoS attacks are well-known application layer attacks.

The studies in  [10,11] used six different ML models including NB, KNN, DT, SVM, RF, and LR on the CICDDoS2019 dataset. Results indicate that the best accuracy of 99% is obtained using the DT and RF models. However, the DT is better than the RF due to lower computational complexity. The authors adopt an image processing-based approach for network attack detection in [3]. The research shows that network traffic transformed into an image can be used with the CNN model for network attack detection. Results using the ResNet model show a 99% accuracy for DDoS attack detection for binary classification and 87% accuracy for eleven kinds of DDoS assaults.

In [5], SVM, KNN, NB, RF, AdaBoost, and XGBoost are used for DDoS detection. Accuracy, F1-score, and training time are used for evaluation. The CICDDoS2019 dataset is used for experiments. XGBoost and AdaBoost are found to accurately predict attacks with 100% accuracy. Another study in [12] implemented RT, KNN, DT, and ANN for DDoS attack detection using the CICDDoS2019 dataset. Results show a 99.95% accuracy for attack detection using the ANN model. Similarly, in [13] the authors employed mathematical and ML models for attack detection using the CAIDA 2007 dataset. The accuracy of LoR varies from 99% to 100%, while NB shows accuracy between 98% and 99%. The results showed an accuracy of 100% for the ML model and an accuracy of 99.75% for mathematical models.

Along the same lines, the study in [14] used eight ML models for DDoS attack detection using the CICIDS2017 dataset. K-fold was used to train algorithms for detecting DDoS attacks. RF was found to be the best algorithm out of eight models. It detects DDoS attacks with 99.885% precision and has 0.05% false alarms. In [15], the authors used gradient descent with momentum algorithm, scaled pooled gradient, and descent algorithm with variable learning rate. An RNN was trained to detect DDoS attacks. The accuracy with variable rate descent algorithm learning is 99.9%. The variable learning rate descent algorithm gave better output than momentum gradient descent and scaled pooled gradient algorithms.

The study in [16] used an RF model together with a highly adaptable neural network algorithm for DDoS attack detection. Results indicate that RF and neural network models achieved 95.2% and 83% accuracy, respectively. Similarly, the authors in [17] proposed a DL model for DDoS detection using RNN in IoT networks. LSTM, Bi-LSTM, and GRU are also used. The proposed models are implemented using the NSLKDD, IoT-NI, IoT-23, BoT-IoT, MQTT, IoT-DS2, and MQTT datasets. Better results are reported for the RNN model. A model called LBDMIDS was proposed in [18] which shows promising performance for intrusion detection. In addition, bidirectional and stacked LSTM are also used for experiments on the UNSW-NB15 and BoT-IoT datasets. Stacked LSTM accuracy was 96.60% and Bi-Directional LSTM accuracy was 96.41% on the UNSW-NB15 dataset. On the BoT-IoT dataset, the accuracy obtained by the bidirectional and stacked LSTM was 99.99%. The results produced by LBDMIDS are the best.

The authors utilize the KDD dataset for experiments using ANN in [19]. The ANN is used with five different algorithms, including Polak–Ribiére conjugate gradient, robust backpropagation, Fletcher–Powell conjugate gradient, variable rate gradient descent algorithm learning, and gradient conjugation with Powell/Beale restarts. Conjugate gradient with Powell/Beale restart showed superior performance, with 99% accuracy. Three different neural networks were compared in [20] for DDoS attack detection. Case cade, feedforward, and fitting neural networks were trained using the one-step secant and Quasi-Newton backpropagation algorithm. Shallow neural gave good accuracy results with less computing time [20].

The study in [21] used SVM to detect DDoS attacks. The authors utilize eight machine learning algorithms, including MLP, LSTM, BiLSTM, KNN, SVM, linear discriminant analysis (LDA), DT, and RF. LSTM and BiLSTM accuracy ranges between 99.9% and 100%. LSTM, MLP, BiLSTM, LDA, KNN, SVM, DT, and RF test accuracy values are 79.5%, 80%, 82.3%, 77%, 82.8%, 69%, 77.7%, and 75.4%, respectively. SVM has better detection of DDoS attack accuracy among ML models, at 97.1%. Results show that BiLSTM performs better among all models. Similarly, the research in [22] used a hybrid model based on RNN extreme learning machine (ELM) algorithms. Features are extracted from the dataset using linear regression with recursive feature extraction and sequence forward selector. For experiments, the NSL-KDD dataset is used. The proposed hybrid model showed enhanced accuracy of up to 99%. Another similar work that utilized the NSL-KDD dataset is [23]. The authors used the LSTM RNN algorithm for detecting DDoS attacks. LSTM achieved a high accuracy of 97.37%.

An LSTM model is used in [24] for DDoS attack detection using the UNSW-NB15 dataset. Binary classification was performed to detect the attack and normal traffic. The model is able to detect attacks with a 99% accuracy and up to 100% precision. The study in [25] combined three algorithms, RNN, LSTM, and CNN, to build a bidirectional CNN-BiLSTM DDoS detection model. The CICIDS2017 dataset was used for evaluating the performance of the proposed model. The individual accuracy of RNN and LSTM reached 99.00%, while CNN showed an accuracy of 98.82%. The proposed CNN-BiLSTM model obtained an accuracy of 99.76%. Similarly, the research [26] utilized the CICDDoS2019 dataset for experiments using a backpropagation neural network called Kalman backpropagation. The model achieved an accuracy of 94% and a precision of 91.22%.

The above-discussed studies indicate that a rich variety of ML models are implemented for DDoS attack detection, including LR, LoR, DT, SVM, NB, KNN, RF, XG Boost, and AdaBoosting. These models are tested on different datasets, such as UNSW-NB15, CICIDS2017, KDD, NSL-KDD, CAIDA 2007, and CICDDOS2019 datasets. While several ML models are tested in the existing studies, DL models are not very well-studied, especially using the CICDDOS2019 dataset. DL methods are much better than ML methods in terms of precision and accuracy, as they can process large amounts of data. This study aims to utilize RNN on CICDDOS2019 to detect DDOS attacks and perform multi-class classification.

## 3. Materials and Methods

**Figure 2:**
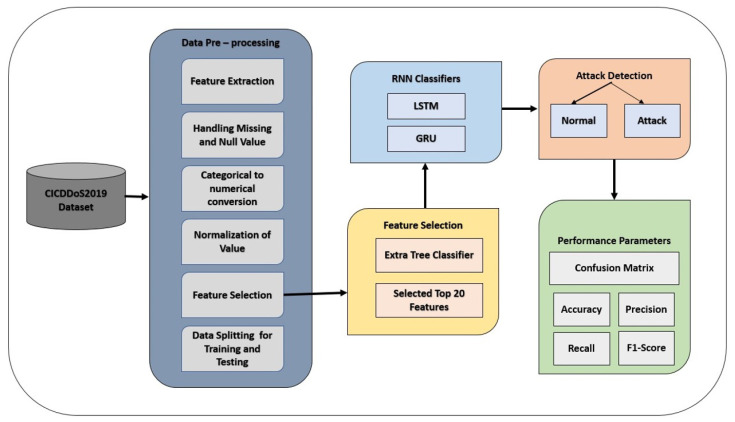
Methodology adopted in this study.

This study proposes an approach based on the RNN model to detect DDoS attacks. In addition, RNN, LSTM, and GRU are used for binary and multi-class classification. Figure 2 illustrates the methodology adopted in the current study. It comprises data normalization, feature extraction, model training, and attack detection modules.

Figure 2 shows that this study uses the CICDDoS2019 dataset for experiments [6]. The dataset must be in an appropriate form for model training to obtain the best performance. For this purpose, data prepossessing is carried out, involving several steps. Missing and null values are removed to reduce ambiguity in data and improve the models’ training process. Categorical values are converted to numerical values as needed by deep learning models. Afterward, the data are normalized. During the data prepossessing step, the feature selection process is carried out to select the top 20 features. The purpose of feature selection is to obtain better performance from the models with less computational complexity. The selected features are the most efficient features for detecting DDoS attacks in network traffic. After that, the data are split into training and testing sub-sets to train RNN, LSTM, and GRU models for binary and multi-classification of attacks. The testing sub-set is later used to test the performance of trained models.

### 3.1. Data Prepossessing

Before training the model, the dataset needs to be preprocessed to remove noise and reduce the amount of redundant or unnecessary data. Data preprocessing is required to improve models’ performance and reduce computational complexity.

#### 3.1.1. Data Normalization

Standard scalar normalization is the process of normalizing the features of the selected dataset for attack detection. The CICDDoS2019 dataset contains different features with different dimensions, scales, and distributions. For example, the ’Fwd Packets/s’ feature contains values that are very large for some records, while very small for others. Utilizing these raw features for training DL models tends to show poor performance. The basic purpose of feature scaling is to ensure that no single feature disproportionately impacts the results. It preserves the relationship between the minimum and maximum values of each feature. So, the features are rescaled into a fixed scale by using standard scalar normalization. Standard scalar normalization used 0 mean and 1 standard deviation for feature rescaling. The expression in (1) is used to obtain a normalized value of the eature [27].

(1) $$x_{n}=\frac{x-\mu }{\sigma }$$

where $x_{n}$ = normalized value, *x* = original value, μ = mean of data, and σ = data standard deviation.

#### 3.1.2. Dealing with Missing and Null Values

As explained above, this study uses the CICDDoS2019 dataset for DDoS attack detection in network traffic. Dealing with missing and null values is an important step in data preprocessing which can impact the accuracy and precision of the models. For the current study, the records that have missing or null values are removed from the dataset. Removing such records reduces computational complexity and improves the performance of the model [28].

#### 3.1.3. Dealing with Categorical Values

ML and DL models work on numerical values, indicating the need to convert categorical values to numerical values. Categorical values and special characters are transformed into numerical values for better model performance. To convert the categorical data types into numerical data types, label encoding and one-hot encoding methods are used. Label encoding converts categorical data into numerical data by assigning a unique numerical label to each category. The sklearn provides a library called LabelEncoder that is used to transform categorical to numerical data [29].

#### 3.1.4. Labels to One-Hot Encoding

When dealing with output labels in the CICDDOS2019 dataset, one-hot encoding is preferable over label encoding since the output labels are categorical and not ordinal in nature. Label encoding gives a unique numeric value to each feature, which implies an inherent ordering of the categories. Label encoding gives a unique numeric value to each class, implying that the classes are inherently ordered. However, there is no meaningful order or link between the distinct classes in the case of output labels. In the case of DDoS attacks, for example, there may be several classes, such as “DrDOS_SNMP”, “TFTP”, “DrDOS_SSDP”, and ICMP, and providing arbitrary numeric values to these classes can bring unexpected associations or biases into the model. This format keeps the label’s categorical characteristics and considers each category as an independent class, with no numerical order or connection imposed. It guarantees that the model understands the categorical characteristics of the output labels and prevents data misinterpretation. As a result, one-hot encoding is recommended for the output labels in the CICDDOS2019 dataset to properly reflect the categorical characteristics of the labels and give a suitable input representation for ML and DL models [30].

#### 3.1.5. Feature Selection

Feature selection is also an important step in data preprocessing. By selecting important and weighed features of the CICDDoS2019 dataset, the attack prediction of the model can be increased. An extra tree classifier is used for feature selection. The extra tree classifier is a decision tree-based classifier, which uses the decision tree approach to select prominent features [31]. For this study, the top 20 features are selected using an extra tree classifier. ’Timestamp’, ’Source Port’, ’Min Packet Length’, ’Fwd Packet Length Min’, ’Flow ID’, ’Packet Length Mean’, ’Fwd Packet Length Max’, ’Average Packet Size’, ’ACK Flag Count’, ’Avg Fwd Segment Size’, ’Fwd Packet Length Mean’, ’Flow Bytess’, ’Max Packet Length’, ’Protocol’, ’Fwd Packetss’, ’Flow Packetss’, ’Total Length of Fwd Packets’, ’Subflow Fwd Bytes’, ’Destination Port’, and ’act_data_pkt_fwd’ are the best 20 features used for model training in this study.

#### 3.1.6. Data Splitting

Data splitting is an important step in data preprocessing too [32]. The CICDDoS2019 dataset is split into training and testing sets. Sklearn library is used for data splitting [33]. A total of 70% of the data are used for training, and 30% are used for testing.

### 3.2. Classification Models

This study used RNN, GRU, and LSTM to detect DDoS attacks. A brief overview of these models is provided for completeness.

#### 3.2.1. Recurrent Neural Network

RNN has several applications, including image processing, market prediction, handwriting recognition, and speech recognition. RNN works better on large amounts of data and using backpropagation improves its final result. The backpropagation vanishing gradient problem arises in RNN and is handled by its variants, the LSTM and GRU models. The RNN is adopted in this study, as the dataset is large and contains attack sequences. When selecting any model for DDoS attack detection, three important factors are considered, including data availability, task complexity, and training resources. Hyperparameter optimization is very important for obtaining optimal performance [34]. The implementation details of the RNN model are provided in Algorithm 1.
**Algorithm 1.** Implementing an RNN model**Require:** Input sequence $x_{1},x_{2},\cdots ,x_{T}$1: Initial hidden state $h_{0}$2: RNN parameters ($W_{xh1},W_{h1h1},W_{h1h2},W_{xh2},W_{h2h2},W_{h2y},b_{h1},b_{h2},b_{y}$)3: Activation function ReLu4: **Decision Making Modules:**5: Attack := 1 & No Attack := 0, T:=0
6: **for all**
t=1
**to**
*T*
**do**7:  hidden states8:  $h1_{0}$ & $h2_{0}\leftarrow$ initialized hidden state $h_{0}$9: **end for**10: Compute the activation of the first RNN layer11:  **if**
$a1_{t}=W_{xh1}\cdot x_{t}+W_{h1h1}\cdot h1_{t-1}+b_{h1}$
**then**12:  Apply the activation function ReLu to the activation13:  Get $a1_{t}\leftarrow$ obtain the hidden state $h1_{t}=\mathrm{ReLU}\left(a1_{t}\right)$14:  Compute the activation of the second RNN layer 15: **else if**
$a2_{t}=W_{h1h2}\cdot h1_{t}+W_{xh2}\cdot x_{t}+W_{h2h2}\cdot h2_{t-1}+b_{h2}$
**then**16:  Apply the activation function ReLU to the activation17:  Get $a2_{t}\leftarrow$ obtain the hidden state $h2_{t}=\mathrm{ReLU}\left(a2_{t}\right)$18: **end if**19: **return** Output of the RNN at time step *t*$y_{t}=W_{h2y}\cdot h2_{t}+b_{y}$ to predict whether incoming traffic is a DDoS attack or not

The parameters of the RNN are represented by various weight matrices and bias vectors in the equations provided. Table 1 provides the details of the symbols used for Algorithm 1.

#### 3.2.2. Long Short-Term Memory

For analyzing network traffic data, LSTM is a better choice than other models. The ability of the LSTM model to recall the previous input helps to find patterns and long-lasting connections at input sequences. The CICDDoS2019 dataset contains details of attacks like flow lengths, source, destination IP, and port number, which shows the sequential nature of attacks in network traffic. LSTM also overcomes the disappearing gradient issue of RNN. In addition, it is used in real-world applications where data are large and data handling is more complicated. LSTM works using an input, output, and forgot gate, which controls the attack flow in and out of cells. Attacks are memorized by the LSTM cell [35]. The LSTM model is trained to classify the instances as normal or attack. The LSTM model has the ability to find the patterns in regular network traffic to detect DDoS attacks. For multi-classification, instances of network traffic values are set to 0, 1, 2, 3. For model training, we used each type of instance from the training dataset of network traffic for proper attack detection type. Label encoding is used to label all attack types and convert the attack to a specific value. The memory cell feature of the LSTM model performs the categorization of network traffic attacks successfully.

#### 3.2.3. Gated Recurrent Unit

The GRU model is also used to detect attacks in network traffic, which takes less memory and is time-efficient. GRU captures long-term relationships in the temporal flow of network traffic. As compared to the RNN and LSTM models, the GRU model is easier to use, which increases computing efficiency without compromising their ability to accurately predict the temporal dynamics in the data. GRU takes less time to train because it has a simplified gate arrangement with no output gate. GRU worked on two sigmoid gates and one hidden state [36]. The GRU model has the ability to find the patterns in regular network traffic to detect DDOS attacks from the CICDDOS2019 dataset.

#### 3.2.4. Hyperparameter Training

For DL models, hyperparameter tuning is the process of determining the optimal combination of various parameters to maximize network performance and efficacy. It entails systematically exploring various hyperparameter values or ranges, training and evaluating the network for each configuration, and selecting the set of hyperparameters that provide the best performance on a validation set or cross-validation. The parameter values of various RNN models vary based on the requirement and dataset. The configuration parameters for model training are displayed in Table 2.

#### 3.2.5. Learning Rate

The learning rate parameter defines the footstep for every repetition as it moves toward the minimum loss function [37]. To find the best learning rate, it is necessary to perform experiments using multiple learning rates. This study used the adaptive moment estimation (Adam) method to find the learning rate for the LSTM and GRU models. The models gave the best optimization at a 0.001 learning rate.

#### 3.2.6. Overfitting Prevention

The overfitting problem occurs during the training of the neural networks. Early stopping and dropout layers are effective methods used in this research for overfitting prevention. To begin, early stopping was used, which allowed the models to run for an additional two rounds before halting to avoid overfitting the training data. Dropout layers were also used, which drop certain neurons at random throughout training to prevent them from dominating the learning process [38].

#### 3.2.7. Activation Functions

This study used rectified linear activation function (ReLU) activation. By applying the ReLU function, the model learned the complicated features of the network’s hidden layers. As compared to other activation functions, like sigmoid and tanh, ReLU results are more efficient [39].

#### 3.2.8. Early Stopping

Early stopping is defined as the technique where the training of the model stops after some time when the performance of the model does not improve after a fixed number of epochs. The early stopping callback keeps track of the validation loss with a 0.001 minimum change. The training will end early if the validation loss does not decrease by at least 0.001 over the course of five consecutive epochs [40].

#### 3.2.9. Optimizer

The Adam optimizer is an optimizing algorithm that uses the RMSprop and AdaGrad techniques. Modifying them in accordance with the first and second moments of the gradients preserves pre-parameter learning rates [41]. The Adam optimizer dynamically modifies the learning rate for each parameter during training to efficiently update the weights of the LSTM and GRU models.

#### 3.2.10. Batch Size

Batch size is defined as the training samples the model takes in each cycle of the training process. As per research, it is found that larger batch size leads to more stable gradients and more stable training models. Smaller batch size leads to the fastest training models but less stable and less accurate models. Batch size typically varies from 32 and above [42]. In the proposed work, the experiments are carried out with batch sizes of 128, 1000, and 2050. A batch size of 1000 gave the best results.

#### 3.2.11. Hidden Layers and Number of Neurons

In this work, 2 LSTM and 2 GRU layers are employed with 2 hidden layers before evaluating the performance of the model. The models were implemented using 8, 128, and 256 neurons, but the results were the same. However, 128 and 256 neurons increased the computational overhead. Therefore, 8 neurons were selected.

### 3.3. Evaluation Matrix

The performance of the models is evaluated using several metrics, including a confusion matrix. There are four parameters of the confusion matrix: true positive (*TP*), true negative (*TN*), false positive (*FP*), and false negative (*FN*).

Accuracy shows how frequently the trained models detect the desired attacks correctly. Accuracy is calculated using

(2) $$Accuracy=\frac{TP+TN}{TP+FN+FP+TN}$$

Precision defines the model’s performance, indicating the TP suggested by the classifier. Precision is defined as the number of TP divided by the total number of positive predictions. It is calculated using

(3) $$Precision=\frac{TP}{TP+FP}$$

The recall of the model is calculated by using the Equation (4).

(4) $$Recall=\frac{TP}{TP+FN}$$

The F1 score is considered a better evaluation parameter, as it combines both precision and recall. We can find the F1 score of the model by using Equation (5).

(5) $$F1\;\;\mathrm{score}=2\times \frac{\left(Precision\times Recall\right)}{\left(Precision+Recall\right)}$$

## 4. Results and Discussion

This section is a crucial part of a research study. It provides a comprehensive review of the major findings, ensuring clarity and brevity by utilizing tables and textual explanations. The overall performance of the proposed methodology is visually represented through graphs, allowing for the identification of patterns in accuracy and loss across different test scenarios. Also, a comparative performance analysis between CICDDoS2019 and CICIDS2017 has been performed. Additionally, to show the superiority of the approach, a comparative table is presented, highlighting the outcomes achieved in comparison with existing state-of-the-art techniques.

### 4.1. Model Implementation

This study utilized RNN, LSTM, and GRU models for DDoS attack identification using the CICDDOS2019 dataset, which is publicly available at [6]. The selected dataset contains thousands of DDoS attacks that fall under 12 classes, including DNS, SNMP, NTP, WebDDoS, MSSQL, UDP, LDAP, NetBIOS, SSDP, PortScan, UDP-Lag, and SYN. This study performs both the binary as well as multi-class classification involving all 12 classes. In all twelve attacks, plans were carried out on the training day, and seven attacks were executed on the testing day; attacks against DNS, SNMP, NTP, WebDDoS, MSSQL, UDP, LDAP, UDP-Lag, NetBIOS, SSDP, SYN, and TFTP were part of the training day, while LDAP, PortScan, MSSQL, UDP-Lag, UDP, and SYN attacks were part of the testing day.

#### Experimental Setup

This study implemented the models using the Python programming language. A Jupyter Notebook was utilized to conduct the experiment. DL application programming interface (API) libraries pandas, matplotlib, sci-kit-learn, Keras, and scipy were used to implement the DL models. A machine with a dedicated GPU of Nvidia 1080Ti with 11 GB of memory was used and took 2 h for model training.

### 4.2. Evaluation Using CICDDoS2019 Dataset

Experiments are performed using the CICDDoS2019 dataset for binary and multi-class classification.

#### 4.2.1. Binary Classification

The CICDDoS2019 [6] dataset offers very encouraging results for binary classification DDoS detection using RNN, LSTM, and GRU, as explained in Table 3.

LSTM and GRU performed well in the intrusion detection assignment on the CICDDOS2019 dataset. They demonstrated high precision, recall, and F1-score, suggesting their usefulness in recognizing and categorizing cyber threats. In terms of execution time, GRU outperformed LSTM, with a much lower execution time of 47.9 s compared to 1 min and 17 s for LSTM and 10 min for RNN. This displays the GRU model’s computational efficiency without compromising performance. GRU’s substantially rapid execution time emphasizes its efficiency as a solution for real-time IDS.

**Figure 3:**
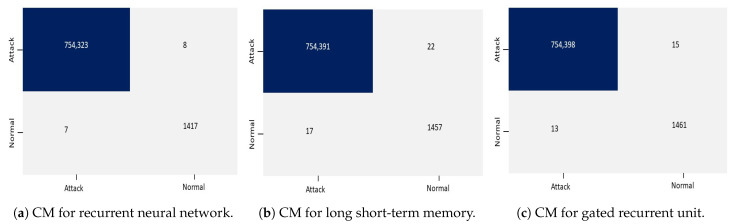
Confusion matrices for binary classification using the CICDDoS2019 dataset.

The confusion matrices in the case of RNN, LSTM, and GRU for binary classification are shown in Figure 3. The results in confusion matrices illustrate that the RNN, LSTM, and GRU models effectively classified a significant number of instances. A huge number of TP cases shows that the models efficiently detected instances of all attacks. Furthermore, the TN instance demonstrates that the models accurately detected the normal instances. LSTM has 22 FP cases and 17 FN instances in terms of misclassifications, whereas GRU has 15 FP cases and 17 FN cases. FN is the number of DDoS assaults that go undetected by the models and are incorrectly classified as normal traffic, whereas FP is the number of instances of normal traffic that are incorrectly classified as DDoS attacks. In order to achieve correct classification in DDoS attack detection, it is crucial to reduce the number of FP and FN cases. Figure 3a demonstrates that only eight instances of normal traffic are FP, which means RNN predicted them as an attack but they actually belong to a normal class. Figure 3b,c demonstrate that 22 by LSTM and 15 by GRU are false positively identified as attacks. Furthermore, seven instances are FN in RNN, indicating that they actually belong to the attack class but are predicted as normal. There were a total of 17 FPs by LSTM and 13 by GRU, which means that RNN has the lowest false positive and false negative rate, but its execution time is higher than LSTM and GRU.

**Figure 4:**
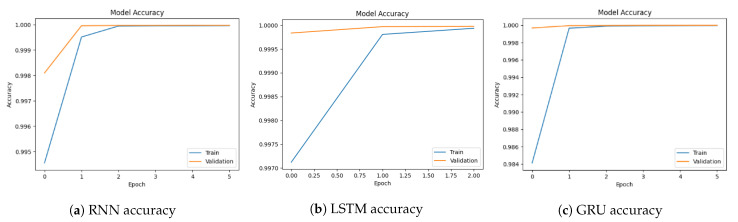
Model accuracy for binary classification using the CICDDoS2019 dataset.

Figure 4a–c show the validation and training accuracy of RNN, LSTM, and GRU. The blue line indicates the training line accuracy and the orange line indicates the validation accuracy. RNN model training accuracy starts from 99.45% and reaches 99.99%. LSTM model training accuracy starts at 99.70% and reaches 99.9%. On the other hand, validation accuracy starts at 99.98% and reaches 99.99%. The GRU model training accuracy starts at 98.4% and reaches 99.99%. This shows that the model effectively learns from the training data and becomes more proficient at making accurate predictions. As the model reaches its highest accuracy, the training accuracy stabilizes, indicating that the model has successfully captured the underlying design in the data and consistently performs well.

**Figure 5:**
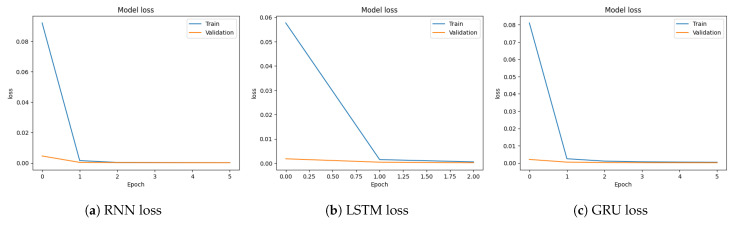
Loss graph for binary classification using the CICDDoS2019 dataset.

Figure 5a–c show the training and validation loss of RNN, LSTM, and GRU. RNN model training loss starts from 0.092, and as the training continues, the loss reaches an impressively low value of 0.0002, indicating that the model accurately fits the data and captures the significant patterns within it. It is the same for LSTM and GRU. LSTM training loss starts from 0.577 and reaches 0.00055. GRU model training loss starts from 0.08 and reaches 0.0004.

Conclusively, in the case of binary classification, the RNN model performs better than LSTM and GRU models by having the fewest FP and FN. This indicates that the RNN model achieved a better balance in accurately identifying positive and negative instances compared to the other models. Furthermore, when examining the loss and accuracy graphs, it can be observed that the RNN model does not exhibit signs of overfitting during training. The validation accuracy and loss of the RNN model are slightly lower than the training accuracy and loss, indicating that the model generalizes well to unseen data. In contrast, the LSTM and GRU models show a slight increase in validation accuracy and loss compared to the training phase, suggesting a higher risk of overfitting. However, LSTM and GRU have faster execution time than RNN. This implies that the LSTM and GRU models may have a tendency to memorize the training data, leading to slightly low performance on unseen data. Overall, the results suggest that the RNN model is more robust and effective in binary classification, as it achieves better accuracy, lower false positive and false negative rates, and shows less risk of overfitting compared to the LSTM and GRU model.

#### 4.2.2. Multi-Class Classification

The CICDDoS2019 dataset offers very encouraging results for DDoS detection using LSTM and GRU for multi-classification, as shown in Table 4.

**Figure 6:**
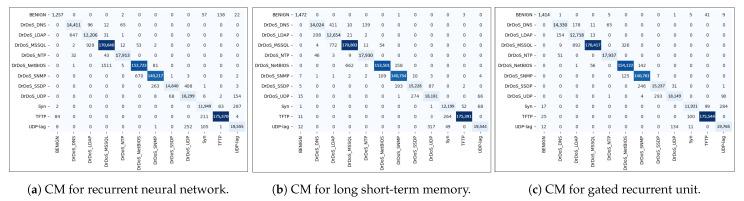
Confusion matrices for multi-classification using the CICDDoS2019 dataset.

Figure 6a shows the confusion matrix for multi-classification in the case of RNN. The analysis of the confusion matrix for the RNN model provided valuable insights into the misclassification patterns. Among all the classes, the class “DrDoS_NTP” has the lowest number of misclassified cases, with only 75 cases being misclassified. However, the class “DrDoS_NETBIOS” has the highest number of misclassified instances, with 1511 cases being wrong, shown as “DrDoS_MSSQL”. This information highlights the specific misclassification tendencies of the model and can help identify areas for improvement or further investigation.

Figure 6b shows the confusion matrix for multi-classification for LSTM. The confusion matrix for LSTM provided insights into the misclassification patterns. It reveals that the classes “Benign” and “DrDoS_NTP” have the lowest number of misclassified instances. Only four cases of the Benign class and fifty-eight cases of the DrDoS_NTP class are misclassified. On the other hand, the classes “DrDoS_MSSQL” and “DrDoS_NETBIOS” have the highest number of misclassified cases. Specifically, 841 instances of the “DrDoS_MSSQL” class are misclassified as “DrDoS_DNS”, “DrDoS_LDAP”, “DrDoS_NTP”, and “DrDoS_ NETBIOS”. Additionally, 772 instances are misclassified as “DrDoS_LDAP”. In the case of the “DrDoS_NETBIOS” class, 662 instances are misclassified as “DrDoS_MSSQL”. Furthermore, 411 instances of the “DrDoS_DNS” class are misclassified as “DrDoS_LDAP”. These misclassification patterns highlight the challenges in accurately distinguishing between certain classes, particularly “DrDoS_MSSQL”, “DrDoS_NETBIOS”, and “DrDoS_DNS”, which exhibit higher rates of misclassification.

Figure 6c shows the confusion matrix for multi-classification for GRU. The confusion matrix of the GRU reveals interesting insights into the classification performance for different classes. It shows that the classes “Benign” and “DrDoS_NTP” have the lowest number of misclassified instances, with 62 instances of “Benign” and 51 instances of “DrDoS_NTP“ being misclassified. This indicates that the GRU model is quite effective in accurately classifying these classes. On the other hand, the classes “DrDoS_MSSQL” and “Syn” have the highest number of misclassified instances. Specifically, 1227 instances of “DrDoS_MSSQL” are misclassified as “DrDoS_LDAP” and “DrDoS_NETBIOS”. Additionally, 892 instances are misclassified as “DrDoS_LDAP” and 326 instances as “DrDoS_NETBIOS”. This illustrates that the GRU model demonstrates more accurate results for identifying and distinguishing instances of the “DrDoS_MSSQL” class. Similarly, 400 instances are misclassified. Among these misclassifications, 284 instances are classified as “UDPLag”, 293 instances of “DrDoS_UDP” are misclassified as “DrDoS_SSDP”, and 246 instances of “DrDoS_SSDP” are misclassified as “DrDoS_SNMP”. These misclassifications highlight the challenges the GRU model faces in accurately differentiating between these classes.

**Figure 7:**
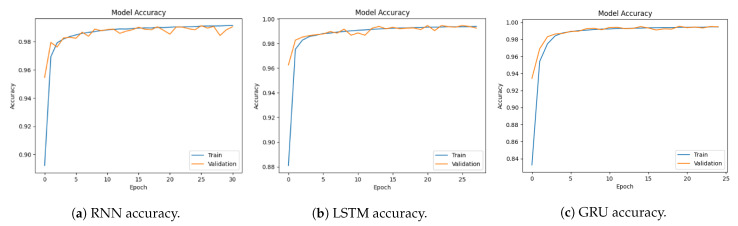
Models’ accuracy for multi-classification using the CICDDoS2019 dataset.

Figure 7a–c show the accuracy of RNN, LSTM, and GRU. The RNN model accuracy starts at 97.5% and reaches 99.15%. The LSTM model accuracy starts at 88% and reaches 99.9%. The GRU model accuracy starts at 83.25% and reaches 99.47%. This indicates the model is gaining knowledge and enhancing its functionality over time.

**Figure 8:**
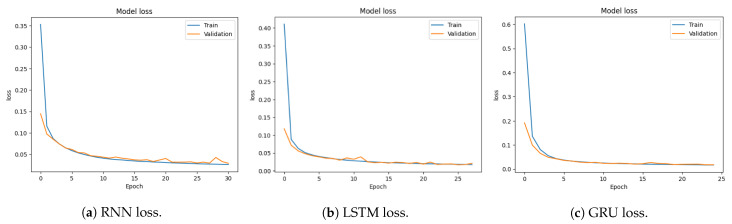
Loss graphs for multi-classification using the CICDDoS2019 dataset.

Figure 8a–c show the training and validation loss of RNN, LSTM, and GRU. The LSTM model loss starts from 0.411 and reaches 0.0176. The GRU model loss starts from 0.60 and reaches 0.0170. Convergence of both accuracy and loss in the training and validation sets demonstrates the effectiveness of the model in learning the underlying patterns of the data and making accurate predictions. The decreasing loss indicates that the model is optimizing its parameters and improving its predictive performance.

In terms of multi-classification, the GRU model outperforms both the LSTM and RNN models, with the fewest misclassified instances. This implies that, as compared to the other models, the GRU model is more effective in correctly classifying instances into their appropriate classes even though the RNN model performed quicker than the GRU model in terms of execution time. When the loss and accuracy graphs of all three models are examined, it is clear that they do not overfit throughout the training procedure. The validation accuracy and loss curves are relatively lower than the training accuracy and loss curves. This shows that the models generalize well to new data and are not impacted by the training data.

### 4.3. Evaluation Using CICIDS2017 Dataset

The results of the DL models are validated through experiments using the CICIDS2017 dataset [43]. Experimental results reveal that the RNN model also detects DDoS attacks in older datasets with greater precision.

#### 4.3.1. Binary Classification

The CICIDS2017 dataset offers very encouraging results for binary classification of DDoS attacks using RNN, LSTM, and GRU models, as given in Table 5. Performance is given in terms of accuracy, precision, recall, F1 score, and execution time. All the models adeptly distinguished between regular and attack activities, with an impressive accuracy of 98% for RNN and LSTM, and 97% for GRU. Every model boasts accuracy, indicating their proficiency in correctly identifying attacks and minimizing false alerts. RNN, LSTM, and GRU all achieved a commendable recall rate of 98% for RNN and LSTM, and 97% for GRU, underscoring their capability to identify false negatives. As for the F1 score, all models performed well. GRU recorded an F1 score of 97%, while LSTM and RNN achieved a 98% F1 score.

**Figure 9:**
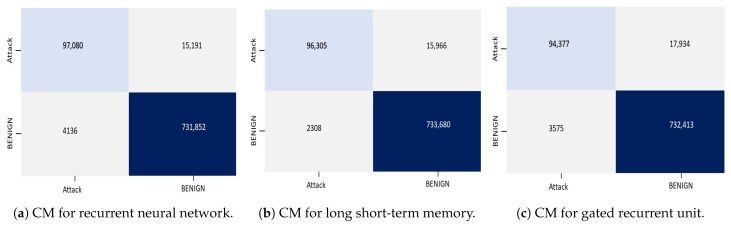
Confusion matrices for binary classification using the CICIDS2017 dataset.

The confusion matrix in the case of RNN is shown in Figure 9a. In this context, the number of TN, 731,852, illustrates the model’s ability to accurately identify benign instances. Conversely, the number of FP, 15,191, points to instances where genuine attacks are mistakenly categorized as benign. Additionally, The FN value of 4136 signifies benign data wrongly classified as attacks, while the TP value of 97,080 indicates successful identification of actual attacks. The confusion matrix in the case of LSTM in Figure 9b shows the model’s performance. It excels in accurately classifying benign instances, with a high value of TN, at 733,680. However, it shows some misclassifications of actual attacks as benign (FN), and there are also instances of benign data being incorrectly classified as attacks (FP). Overall, the model appears to be adept at identifying benign instances but has room for improvement in detecting attacks with higher precision. The confusion matrix in Figure 9c provides insights into the GRU model’s performance. It demonstrates strength in correctly classifying negative class instances, with a high TN value, at 732,413. However, it also shows some misclassifications of actual positive class instances as negative class (FN) and instances of negative class data being incorrectly classified as positive class (FP). This shows that, while the GRU model does a good job of classifying negative class instances, it could do a better job of detecting positive class instances with higher precision. This model appears to be adept at identifying benign instances but has room for improvement in detecting attacks with higher precision.

**Figure 10:**
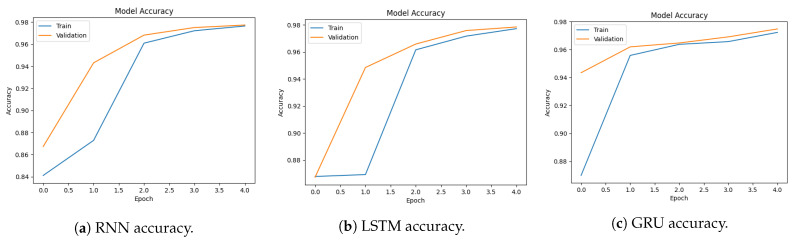
Models’ accuracy for binary classification using the CICIDS2017 dataset.

Figure 10a depicts the progression of the RNN model’s training and validation accuracy. According to the data, the RNN model achieves the highest level of accuracy, at 98%, during the sixth epoch. The training accuracy starts out at 87% and grows gradually to an apex accuracy of 98%. This shows a steady learning curve where the model improves at producing accurate projections. With the increase in the number of epochs, the training consistency is stabilized and the model reaches the optimal accuracy.

The training and validation accuracy is shown in Figure 10b. Results indicate that at the fifth epoch, the maximum accuracy of 98% is attained. The training accuracy trajectory rises gradually from 86% to 98%. The accuracy of the training then stabilizes and stays consistent. On the other hand, the validation accuracy begins at 87% and steadily rises to 98%. The accuracy of the GRU model during training and validation is shown in Figure 10c. At the fifth epoch, the model reaches its maximum accuracy of 99.99%. The training accuracy continually increases from 86% to 97%, demonstrating the model’s efficient learning and prediction ability.

**Figure 11:**
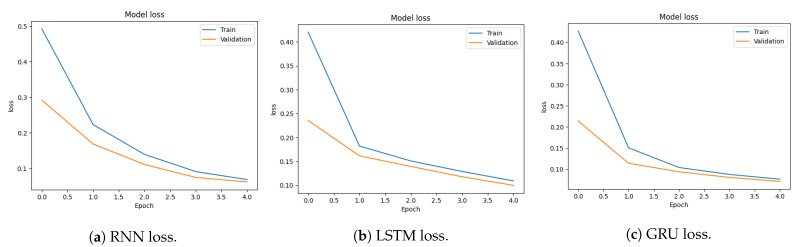
Loss graphs for binary classification using the CICIDS2017 dataset.

Figure 11a depicts the RNN model’s training and validation loss. During the training period, the training loss starts at 0.483 and consistently decreases. This shows how effective the model is at reducing the discrepancy between forecasted and actual values. The loss drops to a noticeably low value of 0.0682 as the training goes on, indicating that the model accurately matches the data and recognizes its key trends.

Figure 11b depicts the convergence of loss through epochs, highlighting the fifth epoch’s lowest loss of less than 0.1091. The training loss starts at 0.4202 and steadily drops to 0.1091. This denotes the model’s enhanced improvement in minimizing the discrepancy between expected and actual values, resulting in a more accurate representation of the data.

The training and validation loss for the GRU is shown in Figure 11c. The training loss starts at 0.4274 and gets smaller with each epoch. This shows how well the model works at closing the gap between expected and actual results. The loss decreases to a negligible 0.0766 over the training period, demonstrating the model’s outstanding data fit and its ability to recognize significant patterns.

#### 4.3.2. Multi-Class Classification

The CICIDS2017 dataset offers very encouraging results for DDoS detection using RNN, LSTM, and GRU for multi-classification, as shown in Table 6. The LSTM and GRU models both displayed outstanding accuracy, precision, F1 score, and recall in the CICIDS2017 dataset, indicating their utility in identifying assaults.

Table 6 lists the performance parameters for each model, including training and testing accuracy as well as recall, precision, and F1 scores. Precision, recall, and F1-score all reached 97% on the LSTM model, which also scored an accuracy of 97%. On the other hand, the GRU model demonstrates an even greater accuracy of 98% and displays a high precision, indicating a lower probability of false positives. A memory and precision balance that is well-maintained is indicated by an F1 score of 97%. The model also displays a remarkable 98% recall rate, indicating that it accurately identified 98% of the attacks. The GRU model outperforms the LSTM model, displaying exceptional performance with an accuracy of 98%. The findings are consistent across all criteria and reflect the same recall, accuracy, and F1-score as LSTM. The execution time for the GRU model is 1 min and 27 s, which is less time than the LSTM model, i.e., 1 min 37 s. Both the LSTM and GRU models show better performance for multi-class intrusion detection using the CICIDS2017 dataset.

**Figure 12:**
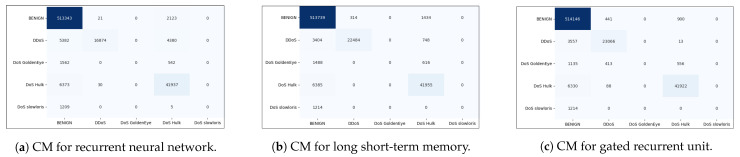
Confusion matrices for multi-class classification using the CICIDS2017 dataset.

Figure 12a–c show the confusion matrices for multi-classification in the case of RNN, LSTM, and GRU, respectively. Figure 12a provides the confusion matrix representing the performance of an RNN model for a multi-class classification problem with five different classes. Each row and column in the matrix corresponds to a specific class, and the numbers in the matrix show how many instances from each true class are classified into each predicted class. A total of 513,739 instances of “Benign” traffic are accurately classified as such, constituting the TPs. However, the model also misclassified 1434 instances of “Benign” traffic as other classes, which are represented as false negatives. The maximum number of false negatives for this class, denoted as 1434, indicates the largest count of instances from the “Benign” class that were incorrectly classified as something else.

The confusion matrix for LSTM in Figure 12b provides insights into the misclassification patterns. In summary, the severity of misclassification for each class depends on the highest count of false negatives or false positives within the confusion matrix. For DDoS attacks, higher misclassification corresponds to 3404 false positives. Conversely, for “Hulk” attacks and “Slowloris” attacks, the most critical misclassification comprises 6385 false negatives for DoS “Hulk” and 6385 false negatives for DoS “Slowloris”. Notably, both DoS “GoldenEye” attacks and DoS “Slowloris” have no correctly classified instances.

In conclusion, the confusion matrix for the GRU model reveals a mixed performance across various classes, as given in Figure 12c. It demonstrates exceptional accuracy in correctly classifying instances of the Benign and DDoS classes, with minimal misclassifications. However, the model faces challenges in distinguishing instances belonging to the DoS “GoldenEye”, DoS “Hulk”, and DoS “Slowloris” classes, resulting in a notable number of misclassifications in these categories.

These findings underscore the strengths and limitations of the GRU model in detecting specific types of DDoS attacks. While it excels in identifying certain attack patterns, further refinements may be necessary to enhance its ability to differentiate between the more intricate attack types. These insights provide valuable guidance for fine-tuning the model and developing strategies to mitigate misclassifications, ultimately improving the accuracy of intrusion detection in diverse network scenarios.

**Figure 13:**
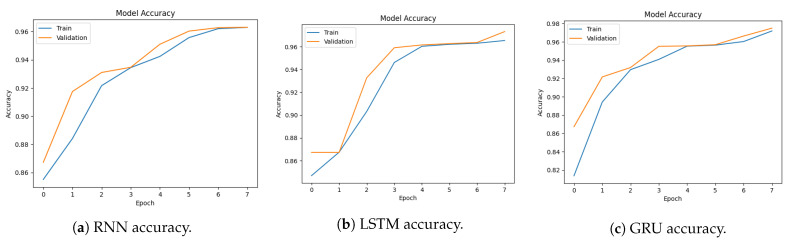
Models’ accuracy for multi-class classification using the CICIDS2017 dataset.

Figure 13a–c show the accuracy of RNN, LSTM, and GRU. The RNN model accuracy starts at 85% and reaches 96%. The LSTM model accuracy starts at 84% and reaches 97%. The GRU model accuracy starts at 81% and reaches 98%.

**Figure 14:**
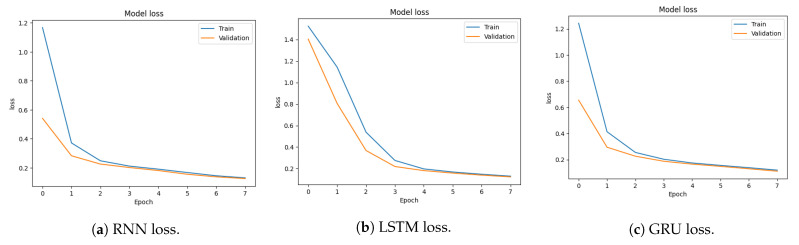
Loss graphs for multi-class classification using the CICIDS2017 dataset.

Figure 14a–c show the training and validation loss of RNN, LSTM, and GRU. The RNN model loss starts from 1.1673 and reaches 0.1300. The LSTM model loss starts from 1.5246 and reaches 0.1293. The GRU model loss starts from 1.2427 and reaches 0.1195.

In terms of multi-classification, the GRU model outperforms both the LSTM and RNN models, with the fewest misclassified instances. This implies that, as compared to the other models, the GRU model is more accurate in effectively classifying instances into their appropriate classes even though the RNN model performs quicker than the GRU model in terms of execution time. When the loss and accuracy graphs of all three models are examined, it is clear that they do not overfit throughout the training procedure. The validation accuracy and loss curves are relatively lower than the training accuracy and loss curves, indicating this. This shows that the models generalize well to new data and are not too impacted by the training data. The GRU model performs better in multi-classification scenarios than the LSTM and RNN models, with the fewest misclassifications. None of the three models overfitted during training, according to an examination of the loss and accuracy graphs for each one. This is supported by the lower validation accuracy and loss measures in comparison to training metrics. It shows that the models generalize well to new data without being significantly influenced by the training dataset.

### 4.4. Comparison with State of the Art

This study performs a comparative analysis of models employed in this study with existing state-of-the-art approaches.

#### 4.4.1. Performance Comparison Using CICDDoS2019 Dataset

The performance of the DL models is compared with existing state-of-the-art methods using the CICDDoS2019 dataset. The employed models aim to improve these state-of-the-art methods for enhancing the accuracy and efficiency of DDoS detection. For this comparison, the best-performing models are compared with other state-of-the-art models’ performance. Table 7 shows the performance comparison of various models that utilized the DDOS19 dataset. Results indicate that the proposed approach in this study tends to show superior results compared to existing models.

#### 4.4.2. Performance Comparison Using CICIDS2017

XGBoost, RF, DT, KNN, CNN, multi-layer perceptron, and LSTM-based approaches have been employed in the existing literature using the CICIDS2017 dataset. Table 8 shows the results for performance comparison. Results indicate that the models employed in this study show superior results on the CICIDS2017 dataset and obtained the highest values for all performance measures. These results show that the proposed approach outperforms other state-of-the-art approaches based on ML and DL models.

#### 4.4.3. Scenario Explanation

The objective of this research is to identify the most suitable model for DDoS attack detection compared to previous research. This study aims to leverage models that are well-suited for analyzing sequential data, as these features are crucial for identifying the patterns and characteristics of DDoS attacks. This research shows the utilization of LSTM, RNN, and GRU models to accomplish this objective. For experiments, this study used the CICDDOS2019 dataset and validated the employed models using the CICIDS2017 dataset, which generates synthetic network traffic comprising both normal and malicious activities. To simulate real-world DDoS attacks, this study deployed a variety of attack strategies, such as SYN flood, UDP flood, and DNS amplification. Prepossessing is used to network traffic data in order to extract relevant features of network flows. These characteristics include packet length, flow time, and protocol type. Following that, the dataset is divided into training and testing sets to evaluate the effectiveness of the LSTM, RNN, and GRU models. We analyzed the classification results, computational efficiency, and robustness to different forms of DDoS attacks of the RNN, LSTM, and GRU models. This study also analyzed previous research studies to see how specific model components affect overall performance [22,23,24,25].

**Figure 15:**
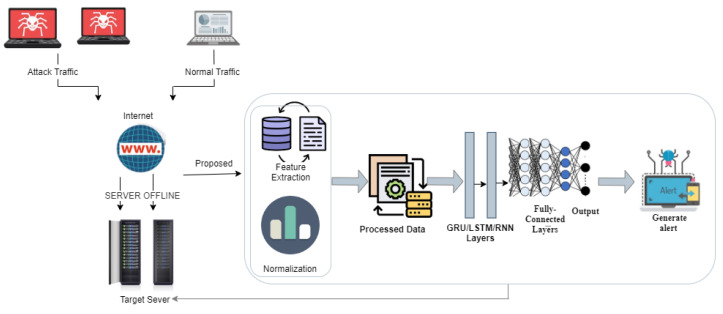
DDoS attack mitigation.

As shown in Figure 15, the performance of the LSTM, RNN, and GRU models is analyzed in the context of detecting DDoS attacks. The classification accuracy, computational efficiency, and resilience to various forms of DDoS attacks are evaluated. Furthermore, a performance review of previous studies is carried out to examine the influence of specific model components on overall performance in order to highlight the importance of choosing the right architecture for DDoS attack detection. The existing literature regarding DDoS attack detection demonstrates high false positives with low precision and low accuracy. This study implemented and analyzed the performance of RNN in particular, as it can identify sequential patterns in network traffic. The results demonstrate that RNN outperforms the other two models for binary classification and GRU outperforms LSTM and RNN for multi-class classification in identifying different types of DDoS attacks.

### 4.5. Comparative Analysis between CICDDOS2019 and CICIDS2017 Datasets

For binary class classification, the accuracy, precision, recall, and F1 score for the RNN and LSTM models are improved from 98% to 99.99% when transitioning from the previous dataset to the CICDDOS2019 dataset. For GRU, the accuracy improves from 97% to 99.99% when transitioning from the previous dataset to CICDDOS2019. So, for RNN and LSTM, the performance improvement on the CICDDOS2019 dataset compared to the previous dataset is approximately 1.99%, while for GRU, it is around 2.99%.

For multi-class classification, RNN, LSTM, and GRU attain 99% accuracy, precision, recall, and F1 score for CICDDOS 2019. Meanwhile, RNN attained 96%, LSTM attained 97%, whereas GRU attained 98% on CICIDS2017. So, for RNN and LSTM, the performance improvement on the CICDDOS2019 dataset compared to CICIDS2017 is approximately 3 and 2%, respectively, while for GRU, it is approximately 1%.

## 5. Conclusions and Future Work

The objective of this research is to detect the DDoS attacks in the latest CICDDOS2019 dataset and validate the model using the CICIDS2017 dataset by employing RNN, LSTM, and GRU. In the proposed work, the RNN, LSTM, and GRU models are evaluated using the top 20 features from the CICDDOS2019 dataset and taking the same features from CICIDS2017. Both models achieved 99% accuracy for both binary and multi-class classification. The RNN model achieves an accuracy of 99.99% for binary classification and 99.54% for multi-class classification, suggesting that it identifies and correctly classifies 99% of all actual positive instances. Overall, the findings indicate that the RNN model is more resilient and successful in binary classification than the LSTM and GRU models, as it achieves higher accuracy, lower false positive and false negative rates, and has a reduced risk of overfitting. For multi-class classification, these findings highlight the superiority of the GRU model in terms of classification performance, while also considering the computational efficiency of the RNN model. The results indicate that the models are able to effectively learn and capture the hidden patterns in data without overfitting, demonstrating their robustness for the detection of different DDoS attacks. Combining rule-based or signature-based techniques with deep learning can help improve the model. Hybrid methods can combine the advantages of both methodologies, allowing for more precise and reliable DDoS attack detection.

## Figures and Tables

**Table 1:** Symbols and their respective descriptions used throughout in Algorithm 1.

Symbol	Description
$a1_{t}$ and $a2_{t}$	The activation function
$W_{xh1}$	Weight matrix for the first RNN layer’s input x_t.
$W_{xh2}$	Weight matrix for the second RNN layer’s input x_t.
$W_{h1h1}$	Weight matrix for the first RNN layer’s previous hidden state $h1_{t-1}$.
$W_{h2h2}$	Weight matrix for the second RNN layer’s previous hidden state $h2_{t-1}$.
$b_{h1}$	The first RNN layer’s bias vector.
$b_{h2}$	Bias vector for the second RNN layer.
$h1_{t}$ and $h2_{t}$	The first and second RNN layers’ hidden states, respectively. They are computed by applying the ReLU activation function to $a1_{t}$ and $a2_{t}$.
$y_{t}$	The output
$W_{h2y}$	Weight matrix for the hidden state $h2_{t}$ to the output $y_{t}$.
$b_{y}$	Bias vector for the output.

**Table 2:** Parameters for RNN, LSTM, and GRU models.

Parameters	LSTM	GRU
Activator	Relu, Softmax (multiclass), Sigmoid (binary class)	Relu, Softmax (multiclass), Sigmoid (binary class)
Optimizer	Adam	Adam
Learning rate	0.001	0.001
Loss	Categorical cross entropy (multiclass), Binary cross entropy (binary-class)	Categorical cross entropy (multiclass), Binary cross entropy (binary-class)
LSTM/GRU layers	2	2
Hidden layers	2	2
Neurons per LSTM layers	8	8
Neurons per hidden layers	16, 8 (1st Layer, 2nd layer)	16, 8 (1st Layer, 2nd layer)
Batch size	1000	1000
Epochs	100	100

**Table 3:** Performance results for binary classification using the CICDDoS2019 dataset.

Performance Measure	RNN	LSTM	GRU
Accuracy	99.99%	99.99%	99.99%
Precision	99.99%	99.0%	99.0%
Recall	99.99%	99.0%	100%
F1 score	99.99%	99.0%	100%
Execution time	10 min	1 min 17 s	47.9 s

**Table 4:** Experimental results for multi-classification using the CICDDoS2019 dataset.

Performance Measures	RNN	LSTM	GRU
Accuracy	99.15%	99.43%	99.54%
Precision	97%	98%	98%
Recall	97%	99%	99%
F1-score	97%	98%	98%
Execution time	4 min	16 min 30 s	7 m 3 s

**Table 5:** Binary classification results for the CICIDS2017 dataset.

Performance Measures	RNN	LSTM	GRU
Accuracy	98.0%	98.0%	97.0%
Precision	98.0%	98.0%	97.0%
Recall	98.0%	98.0%	97.0%
F1-score	98.0%	98.0%	97.0%
Execution time	1 min 27 s	1 min 18 s	1 min 30 s

**Table 6:** Results for multi-class classification using the CICIDS2017 dataset.

Performance Measures	RNN	LSTM	GRU
Accuracy	96.0%	97.0%	98.0%
Precision	96.0%	97.0%	97.0%
Recall	96.0%	97.0%	97.0%
F1-score	96.0%	97.0%	97.0%
Execution time	1 min 42 s	1 min 37 s	1 m 27 s

**Table 7:** Performance comparison with state of the art using the CICD-DoS2019 dataset.

SDN Methods	Precision	Recall	F1-Score	Accuracy
ResNet	80%	38%	51%	87%
Naïve Bayes	51%	49%	49%	57%
Random Forest	78%	70%	73%	86%
Decision Tree	92%	60%	40%	77%
Logistic Regression	86%	11%	19%	95%
Neural Network	79%	4%	53%	83%
Hybrid Model	80%	72%	75%	95%
SVM	29%	7%	11%	97%
MLP	72%	11%	19%	79%
KNN	61%	4%	48%	77%
LSTM for binary classification	99.0%	99.0%	99.0%	99.99%
GRU for binary classification	99.0%	100%	100%	99.99%
LSTM for Multi classification	98%	99%	98%	99.43%
GRU for Multi classification	98%	99%	98%	99.54%

**Table 8:** Performance comparison with state of the art using the CICIDS2017 dataset.

SDN Methods	Precision	Recall	F1-Score	Accuracy
KNN	76%	67%	74%	70%
Deep Neural Network	87%	81%	74%	77%
Decision Tree	84%	86%	76%	86%
Multi-Layer Perceptron	72%	79%	68%	73%
XGBoost	84%	73%	83%	78%
CNN	89%	86%	79%	86%
LSTM	91%	91%	92%	90%
Random Forest	64%	67%	82%	74%
LSTM for binary classification	98%	98%	98%	98%
GRU for binary classification	97%	97%	97%	97%
LSTM for Multi classification	97%	97%	97%	97%
GRU for Multi classification	98%	97%	98%	97%

## Data Availability

Not applicable.
